# Peripheral Stent Thrombosis Leading to Acute Limb Ischemia and Major Amputation: Incidence and Risk Factors in the Aortoiliac and Femoropopliteal Arteries

**DOI:** 10.1007/s00270-016-1513-0

**Published:** 2016-12-05

**Authors:** Konstantinos Katsanos, Said A. M. Al-Lamki, Aneeta Parthipun, Stavros Spiliopoulos, Sanjay Dhanji Patel, Ioannis Paraskevopoulos, Hany Zayed, Athanasios Diamantopoulos

**Affiliations:** 1 Department of Interventional Radiology, Imaging Sciences Division, Guy’s and St. Thomas’ Hospitals, NHS Foundation Trust, King’s Health Partners, London, SE1 7EH UK; 2 Department of Radiology, The Royal Hospital, PC 121, 685 Muscat, Oman; 3 2nd Department of Radiology, Interventional Radiology Unit, ATTIKO Athens University Hospital, 1st Rimini St, Chaidari, 12461 Athens, Greece; 4 Academic Department of Surgery, Cardiovascular Division, Guy’s and St. Thomas’ Hospitals, NHS Foundation Trust, King’s Health Partners, London, SE1 7EH UK

**Keywords:** Nitinol stent, Covered stent, Stent thrombosis, Acute limb ischemia, Major amputation

## Abstract

**Purpose:**

To report the real-world incidence and risk factors of stent thrombosis in the aortoiliac and femoropopliteal arteries in case of bare nitinol stent (BNS) or covered nitinol stent (CNS) placement from a single-centre retrospective audit.

**Materials and Methods:**

Medical records of consecutive patients treated with peripheral stent placement for claudication or critical limb ischemia were audited for definite stent thrombosis defined as imaging confirmed stent thrombosis that presented as acute limb-threatening ischemia. Cases were stratified between aortoiliac and femoropopliteal anatomy. Cox regression analysis was employed to adjust for baseline clinical and procedural confounders and identify predictors of stent thrombosis and major limb loss.

**Results:**

256 patients (*n* = 277 limbs) were analysed over a 5-year period (2009–2014) including 117 aortoiliac stents (34 CNS; 12.8 ± 5.0 cm and 83 BNS; 7.8 ± 4.0 cm) and 160 femoropopliteal ones (60 CNS; 21.1 ± 11.0 cm and 100 BNS; 17.5 ± 11.9 cm). Median follow-up was 1 year. Overall stent thrombosis rate was 6.1% (17/277) after a median of 43 days (range 2–192 days) and affected almost exclusively the femoropopliteal segment (12/60 in the CNS cohort vs. 4/100 in the BNS; *p* = 0.001). Annualized stent thrombosis rates (per 100 person-years) were 12.5% in case of CNS and 1.4% in case of BNS (HR 6.3, 95% CI 2.4–17.9; *p* = 0.0002). Corresponding major amputations rates were 8.7 and 2.5%, respectively (HR 4.5, 95% CI 2.7–27.9; *p* = 0.0006). On multivariable analysis, critical leg ischemia and CNS placement were the only predictors of stent thrombosis. Diabetes, critical leg ischemia, femoropopliteal anatomy, long stents and CNS were independent predictors of major amputations.

**Conclusions:**

Placement of long femoropopliteal covered nitinol stents is associated with an increased incidence of acute stent thrombosis and ensuing major amputation. Risks are significantly lower in the aortoiliac vessels and with use of bare nitinol stents.

## Introduction

Peripheral arterial disease (PAD) has a rapidly increasing incidence and the projected number of patients affected globally has increased from 164 million to 202 million between 2000 and 2010, demonstrating a nearly 25% over 10 years, while up to 3% of these patients are expected to develop critical limb ischemia (CLI), the final stage of PAD that is typically characterized by high risk of cardiovascular death and lower limb amputation [1]. Treatment options for symptomatic PAD include conservative medical therapy, open surgical and more often percutaneous, minimally invasive, peripheral endovascular procedures. The latter are recommended as first line revascularization strategy in most of the cases, constantly gaining ground compared to surgical therapies [2–4]. This could be mainly attributed both to the growing experience in challenging peripheral interventions and also to technological developments of safer and more efficient endovascular devices, such as low-profile nitinol stents [5, 6].

According to a recent mixed treatment meta-analysis of level I evidence, use of bare metal, covered and drug-eluting nitinol stents results in superior technical success and patency rates compared to traditional plain balloon angioplasty [7]. Although use of nitinol stents has been established in everyday clinical practice for the management of iliac or femoropopliteal arterial disease, peripheral stent re-occlusion remains a frequent phenomenon, leading to symptomatic recurrence and repeat interventions in the majority of the cases [8]. Still, very little is known about acute limb ischemia and major amputations following development of acute peripheral stent thrombosis [9, 10].

Stent occlusion differs fundamentally from acute thrombosis both in mechanism and clinical presentation. While stent occlusion occurs following slow progressive in-stent restenosis because of neointimal hyperplasia, which allows for collateral formation, thrombosis is an acute phenomenon occurring immediately, soon after stent deployment (acute thrombosis), or even later (late or very late stent thrombosis) [11]. Depending on the timing and the available collateral network, stent re-occlusion because of restenosis may even go undetected, whereas stent thrombosis will usually present as limb threatening, acute limb ischemia (ALI) at high risk of amputation [10]. We sought to investigate the actual incidence, risk factors and clinical implications of the phenomenon of stent thrombosis in the aortoiliac and infrainguinal arteries in cases of covered nitinol stent (CNS) or bare nitinol stent (BNS) placement.

## Materials and Methods

This was a single-centre, retrospective clinical audit which did not require Institutional review board ethics approval as per National Health Service Research and Ethics definitions (Institutional Review Board equivalent, http://www.nres.nhs.uk/). Patient archives from a high-volume tertiary teaching centre were audited for cases of stent thrombosis resulting in acute limb ischemia. The study searched the medical records of all patients treated with percutaneous bail-out, primary, or direct, bare nitinol metal or covered nitinol stent (stent-graft) deployment at the aortoiliac and/or femoropopliteal arteries between January 2009 and December 2014 to allow for adequate follow-up to date. Hospital records were analysed in depth and patient baseline demographics, clinical presentation at baseline, procedural and lesion characteristics, any intra-procedural and post-procedural complications as well as follow-up visits and repeat procedures were recorded. Completion angiogram of the index procedure was used to evaluate infrapopliteal run-off vessels and a simplified 0–2 score was used for run-off quantification, as previously reported [12]. All stent placement procedures were performed or supervised by nine interventional radiology or vascular surgery consultants with a median of 5 years of experience in peripheral arterial interventions (range 3–20 years). Stent choice was based on operator’s preference and governed by patient-specific clinical and anatomical criteria as part of real-world standards of endovascular care. At the time of implantation (2009–2014), there was little comparative evidence to support one type of stent over the other, but empirically, covered stents were usually chosen in cases of chronic total occlusions and lesions with predominantly thrombotic or heavily calcified elements in order to prevent against distal vessel embolization. Discharge medications and follow-up outpatient clinical appointments were also audited. A loading dose of Clopidogrel 300 mg was routinely given if the patient had not been on regular Clopidogrel prescription before, and dual antiplatelet therapy with Aspirin 75 mg and Clopidogrel 75 mg once daily was advised post-procedure for a duration of up to 6 months per local Trust policy. Colour-doppler ultrasound scans, medical notes relevant to the acute admission of recurrent ischemia and digital subtraction angiograms during subsequent attempts of transcatheter thrombolysis and/or other re-interventions were examined in detail to confirm stent thrombosis. Data analysis was performed by an interventional radiology fellow and an interventional radiology consultant (7 years of experience) in consensus.

Type of stent used during the index procedure depended solely on the operator’s preference. CNS stents used in this study was the GORE^®^ VIABAHN^®^ Endoprosthesis (W. L. Gore & Associates (UK) Ltd, Livingston, UK), while a variety of BNS stents were placed including mainly the Zilver 635^®^ or Zilver PTX Vascular Self-Expanding Stents (COOK Medical Europe LTD, Limerick, IRELAND), the GORE^®^ TIGRIS^®^ Vascular Stent (W. L. Gore & Associates (UK) Ltd, Livingston, UK) and the SUPERA^®^ Peripheral Stent System (Abbott Vascular, IL, USA). Baseline demographics and procedural details are outlined in detail reported in Tables 1 and 2.

**Table 1:** Patient demographics

	Covered nitinol stents	Bare nitinol stents	*p* value
Patients (*n*)	91	165	–
Limbs (*n*)	94	183	–
Age (years)	73.9 ± 11.8	69.9 ± 11.0	0.006
Diabetes	24/91 (26.4)	56/165 (33.9)	0.21
Smoking	37/91 (40.6)	106/165 (64.2)	0.0003
Hypertension	57/91 (62.6)	72/165 (43.6)	0.0004
Hyperlipidemia	51/91 (56.0)	63/165 (38.2)	0.0006
Chronic kidney disease	33/91 (36.2)	44/165 (26.6)	0.11
Critical limb ischemia	61/94 (67.0)	75/183 (41.0)	0.0002
Lesion anatomy			
Aortoiliac vessels	34/94 (36.2)	83/183 (45.3)	0.14
Aortoiliac diameter	8.5 ± 0.9 mm	8.6 ± 0.9 mm	0.59
Infrainguinal vessels	60/94 (63.8)	100/183 (54.6)	0.14
Infrainguinal diameter	5.2 ± 0.4 mm	5.1 ± 0.4 mm	0.13
Lesion mode			
Arterial stenosis	16/94 (17.0)	97/183 (53.0)	0.0001
Arterial occlusion	68/94 (72.3)	86/183 (47.0)	0.0001
Acute vessel occlusion	6/94 (6.4)	5/183 (2.7)	0.19
Chronic total occlusion	62/94 (65.9)	81/183 (44.2)	0.0006
Vessel rupture	10/94 (10.6)	0/183 (0)	0.0001

Categorical data are given as counts and percentages in the parentheses

Data presented as mean ± standard deviation (SD)

**Table 2:** Procedural variables

	Covered nitinol stents	Bare nitinol stents	*p* value
Treatment mode			
Subintimal angioplasty	56/94 (59.6)	80/183 (43.7)	0.006
Intraluminal angioplasty	38/94 (40.4)	103/183 (56.3)	0.006
Number of stents	1 (1–2)	1 (1–2)	0.99
Overlapping stents	29/94 (30.8)	57/183 (31.1)	1.00
Stented lesion length^a^	18.1 ± 10.1 cm	13.0 ± 10.4 cm	<0.0001
Stent length (range)	2–46 cm	5–40 cm	–
Aortoiliac length^a^	12.8 ± 5.0 cm	7.8 ± 4.0 cm	<0.001
Aortoiliac stent diameter^a^	8.3 ± 0.8 mm	8.2 ± 0.7 mm	0.50
Infrainguinal length^a^	21.1 ± 11.0 cm	17.5 ± 11.9 cm	<0.001
Infrainguinal stent diam^a^	5.8 ± 0.5 mm	6.1 ± 0.4 mm	<0.0001
Run-off arteries^b^	2 (1–2)	2 (1–3)	0.06
Antiplatelet therapy			
None	3/94 (3.2)	15/183 (8.2)	0.13
Aspirin	23/94 (24.4)	61/183 (33.3)	0.13
Clopidogrel	7/94 (7.4)	21/183 (11.5)	0.29
Dual antiplatelet	54/94 (57.4)	83/183 (45.3)	0.06
Warfarin	7/94 (7.4)	3/183 (1.6)	0.03

Categorical data are given as counts and percentages in the parentheses

^a^Data presented as mean ± standard deviation (SD)

^b^Data presented as median and interquartile range (IQR) in the parentheses

The primary outcome measure was the incidence of definite acute stent thrombosis. Similar to the ARC classification system of coronary stent thrombosis [13], we defined definite peripheral stent thrombosis as imaging confirmed (Duplex ultrasonography and/or digital angiography) thrombotic occlusion of CNS or BNS presenting with acute limb ischemia (ALI) symptoms. ALI was defined as a sudden decrease in limb perfusion causing a potential threat to limb viability, according to standard clinical criteria of international guidelines [14]. Secondary endpoints included comparison of major amputation rates between CNS and BNS and analysis of primary patency of the implanted stents. The latter was defined as uninterrupted patency of the stent demonstrated by duplex follow-up and/or clinical and hemodynamic criteria demonstrating maintenance of achieved improvement with no additional procedures performed on or at the margins of the stent [15]. Major amputations were defined as any limb loss extending above the ankle level. Cases were stratified between aortoiliac and femoropopliteal anatomy. We further looked for any correlation between thrombotic and/or major amputation events with baseline anatomical, clinical and procedural variables in order to identify independent predictors of leg amputations and stent thrombosis.

### Statistical Analysis

Correlation of stent thrombosis and major amputation events with baseline anatomical and clinical variables was first performed within a univariate framework. Next, Cox regression multivariable analysis was performed to further adjust for confounders and identify independent predictors of stent failure and limb loss. Dependent variables in the Cox model included a smoking habit, hypertension (prescribed antihypertensive drug therapy), hyperlipidemia (abnormal blood lipid levels, or prescribed drug therapy), chronic kidney disease (serum creatinine levels >1.5 mg/dl or dialysis), diabetes mellitus (drug therapy or insulin), baseline symptoms (critical leg ischemia vs. intermittent claudication), treatment site (aortoiliac vs. infrainguinal lesions), stented lesion length (dichotomized at the median value), baseline lesion type (chronic total occlusions vs. stenosis), stent type (CNS vs. BNS) and prescribed antiplatelet regiment (dual- vs. mono-therapy). Results were expressed as Hazard ratios (HRs) with associated 95% confidence intervals (95% CIs) as indicated for time-to-event analyses. Kaplan–Meier survival analysis was implemented to demonstrate the incidence of stent thrombosis and major amputations over time. Events were stratified between aortoiliac and femoropopliteal anatomy. Discrete variables are presented as counts and percentages, while continuous variables as medians and interquartile range in parentheses, or as means ± standard errors (SE) if originating from normal distributions. Statistical analysis was performed with the GraphPad Prism statistical software package (version 5; GraphPad Software, La Jolla, CA, USA). The Cox model was performed with the Statsdirect statistical package (version 2.7.9; Statsdirect Ltd, Altrincham, UK). The threshold of statistical significance was set at *p* < 0.05 for all comparisons. The lead author was responsible for the statistical analysis.

## Results

### Patient Cohort

In total, 256 patients with 277 limbs were analysed with a median of 1 (IQR 1–2) overlapping stents placed in each segment per limb. In total, we evaluated 117 aortoiliac stented segments (34/117; 29.0% CNS and 83/117; 71.0% BNS) and 160 femoropopliteal stented segments (60/160; 37.5% CNS and 100/160; 62.5% BNS) (Table 2). The BNS group included 14 limbs treated with the ZILVER-PTX drug-eluting stent. There were no cases of drug-coated balloon application in combination with CNS or BNS stent placement. The majority of the patients were treated due to chronic atherosclerotic disease (96%), while 11/277 cases (4.0%) involved a background of acute vessel occlusions (6/94; 6.4% with covered stents and 5/183; 2.7% with bare stents; *p* = 0.19). Median follow-up period available was 1 year (389 person-years analysed).

Baseline comorbidities were more pronounced in the CNS cohort that included significantly older (73.9 ± 11.8 vs. 69.9 ± 11.0; *p* = 0.006), more hypertensive (62.6 vs. 43.6%; *p* = 0.0004), more hyperlipidemic (56.0 vs. 38.2%; *p* = 0.0006) and more CLI patients (67.0 vs. 41.0%; *p* = 0.0002). On the other hand, the BNS included significantly more smokers (64.2 vs. 40.6%; *p* = 0.0003). In addition, significantly more occlusions (72.3 vs. 47.0%; *p* = 0.0001), longer segments (18.1 ± 10.1 vs. 13.0 ± 10.4 cm; *p* < 0.0001) and more cases of subintimal recanalization (59.6 vs. 43.7%, *p* = 0.006) were included in the CNS cohort. There were ten cases of vessel rupture which were treated with bail-out CNS (10/94; 10.6% vs. 0/183 in the BNS group; 0%; *p* = 0.0001).

There were no significant differences between the two cohorts with regard to antiplatelet therapy and run-off arteries on the completion angiogram of the index procedure (Table 2). Single antiplatelet therapy with aspirin or Clopidorel was prescribed in 31.8% in the CNS group compared to 44.8% in the BNS arm (*p* = 0.20); dual antiplatelet was prescribed in 57.4% of the CNS cases versus 45.3% in the BNS arm (*p* = 0.06).

### Peripheral Stent Thrombosis

Cumulative primary patency rate was 70.2% for CNS and 72% for BNS (*p* = 0.72), after a median follow-up period of 342 days (range 1–913) and 440 days (range 1–2493), respectively. Cumulatively, stent thrombosis was documented in 6.1% (17/277 limbs) after a median of 43 days (range 2–192 days) and was significantly higher in the CNS cohort (13/94; 13.8% vs. 4/183; 2.2% in the BNS group; *p* = 0.0003). In the BNS group, 1 stent thrombosis occurred in a ZILVER-PTX case. All stent thromboses involved the femoropopliteal segment with the exception of 1 aortoiliac CNS thrombosis (long common-external iliac occlusion). All stent thromboses occurred in patients with chronic peripheral occlusive disease and there was no recurrent thrombosis noted in the subgroup of acute vessel occlusions at baseline (17/266 vs. 0/11; *p* = 1.00).

**Fig. 1:**
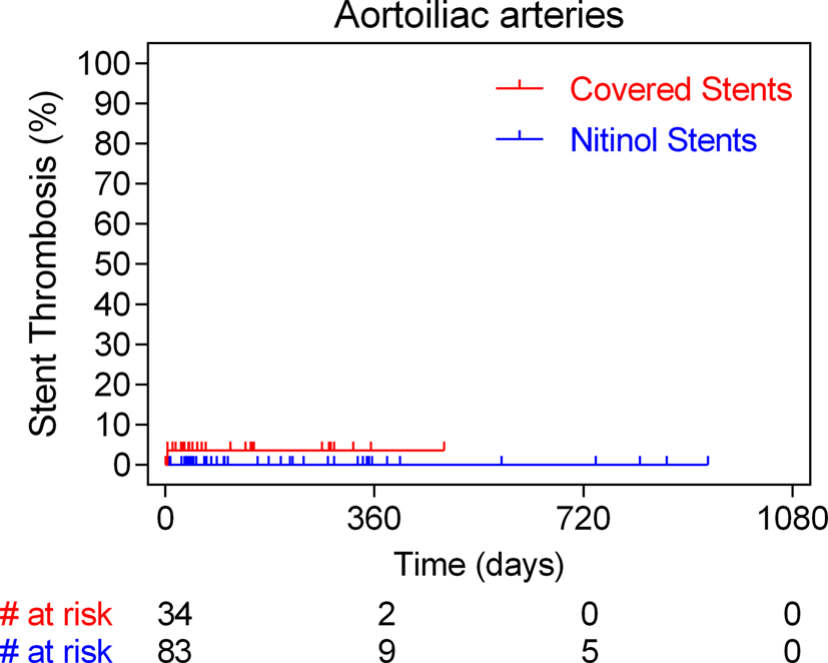
Time-to-event analysis with the Kaplan–Meier method of events of stent thrombosis in case of aortoiliac arteries stratified according to the stent type used (covered and bare nitinol stent cohorts)

**Fig. 2:**
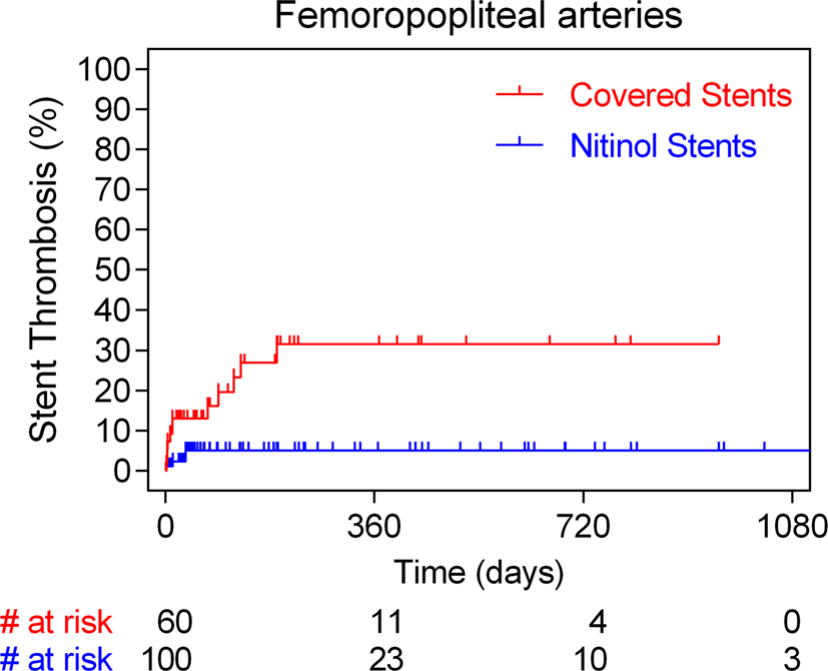
Time-to-event analysis with the Kaplan–Meier method of events of stent thrombosis in case of femoropopliteal arteries stratified according to the stent type used (covered and bare nitinol stent cohorts)

Annualized event rate (per 100 person-years) of acute stent thrombosis was 12.5% in case of covered stents and 1.4% in case of bare nitinol ones (HR 6.3, 95% CI 2.4–17.9; *p* = 0.0002). The majority of the thirteen CNS thromboses were noted within the first month after the intervention, while no acute thrombotic events were noted after 6 months follow-up. On the contrary, all four BNS thromboses occurred within the first month post-deployment. Time-to-event analysis of all stent thrombosis in the aortoiliac and femoropopliteal arteries is shown in Figs. 1 and 2, respectively. On multivariable Cox regression analysis, critical limb ischemia (HR 6.0, 95% CI 1.26–28.9; *p* = 0.02) and use of CNS (HR 3.4, 95% CI 1.02–11.2; *p* = 0.04) were found to be significant independent predictors of stent thrombosis (Table 3).

### Major Limb Amputations

**Fig. 3:**
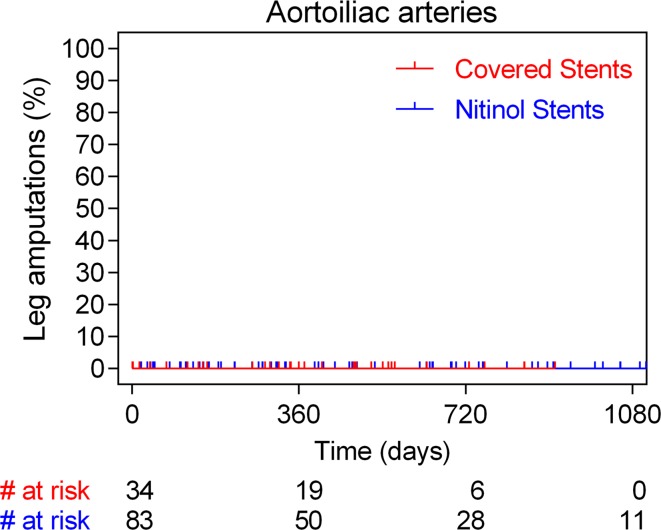
Time-to-event analysis with the Kaplan–Meier method of major lower limb amputations in case of aortoiliac arteries stratified according to the stent type used (covered and bare nitinol stent cohorts)

**Fig. 4:**
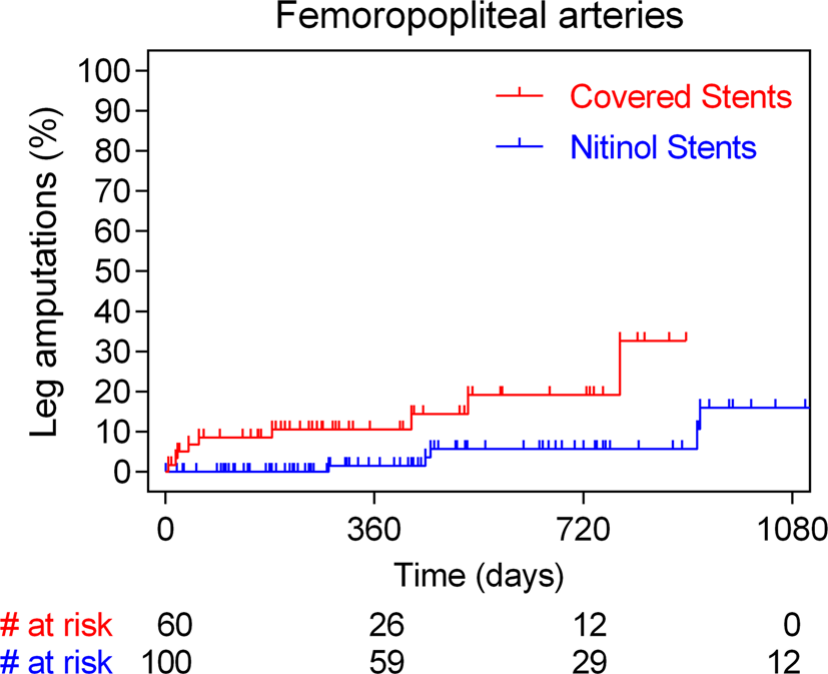
Time-to-event analysis with the Kaplan–Meier method of major lower limb amputations in case of femoropopliteal arteries stratified according to the stent type used (covered and bare nitinol stent cohorts)

Treatment mode of the thrombosed cases presenting with ALI and detailed event rates are presented in Table 4. Most cases were addressed with catheter-directed thrombolysis or vein bypass surgery in case of contra-indication to receive thrombolysis (Table 3). All major amputations occurred in patient treated with femoropopliteal stent placement—there were no major amputations documented in the aortoiliac group. Within the setting of acute stent thrombosis and ALI presentation, four major amputations occurred within the first 2 months exclusively in the CNS cohort (4/94; 4.2% vs. 0/183; 0.0% in the BNS; *p* = 0.01). All four cases of BNS thrombosis were successfully treated (three percutaneous catheter-directed thrombolysis and one surgical vein bypass). Corresponding stent-thrombosis-related amputation rates were 3.9 and 0.0%, respectively. Cumulatively, more major amputations were noted following CNS placement compared to BNS use. Long-term annualized rates of lower limb loss (per 100 person-years) were 8.7 and 2.5%, respectively (HR 4.5, 95% CI 2.7–27.9; *p* = 0.0006). Time-to-event analysis of all major amputations in the aortoiliac and femoropopliteal arteries is shown in Figs. 3 and 4, respectively. Multivariable Cox regression analysis showed that diabetes (HR 9.9, 95% CI 2.28–43.5; *p* = 0.002), critical limb ischemia (HR 10.5, 95% CI 1.80–61.3; *p* = 0.009), long stented segments (HR 15.1; 95% CI 2.0–114.1; *p* = 0.008), femoropopliteal anatomy (HR 22.4, 95% CI 1.00–518.7; *p* = 0.05) and CNS placement (HR: 7.3; 95% CI 1.41–38.0; p = 0.02) were independent predictors of major amputations (Table 5).

**Table 3:** Univariate and multivariate analysis for stent thrombosis

	Univariate analysis HR (95% CI)	Multivariate analysis HR (95% CI)	*p* value
Smoking	1.34 (0.52–3.47)	1.08 (0.37–3.09)	0.89
Hypertension	1.05 (0.37–2.99)	0.87 (0.27–2.83)	0.82
Hyperlipidemia	1.53 (0.56–4.15)	1.11 (0.33–3.67)	0.87
Chronic kidney disease	0.95 (0.34–2.71)	0.75 (0.25–2.23)	0.61
Diabetes mellitus	2.28 (0.88–5.92)	1.55 (0.51–4.66)	0.44
Critical limb ischemia	9.56 (2.18–41.9)	6.03 (1.26–28.9)	0.02
Femoropopliteal anatomy	9.64 (1.28–72.7)	5.54 (0.66–46.6)	0.11
Stented lesion length	4.79 (1.38–16.7)	1.86 (0.43–8.13)	0.41
Chronic total occlusions	3.73 (1.07–13.0)	1.95 (0.45–8.39)	0.37
Stent grafts	6.27 (2.04–19.2)	3.38 (1.02–11.2)	0.04
Dual antiplatelet agents	0.91 (0.34–2.46)	1.00 (0.33–3.06)	0.99

Categorical data are given as counts and percentages in the parentheses

^a^Data presented as median and range in the parentheses

**Table 4:** Primary and secondary outcome measures

	Covered nitinol stents	Bare nitinol stents	*p* value
Follow-up (days)^a^	342 (1–913)	440 (1–2493)	–
Follow-up (person-years)	104.0	284.8	–
Stent primary patency	66/94 (70.2)	132/183 (72.1)	0.74
Stent thrombosis	13/94 (13.8)	4/183 (2.2)	0.0003
Event rate (per 100 person-years)	12.5%	1.4%	–
Treatment mode			
Catheter thrombolysis	8/13 (61.5)	3/4 (75.0)	1.00
Successful thrombolysis	5/13 (38.4)	3/3 (100)	0.04
Surgical bypass	3/13 (23.1)	1/4 (25.0)	1.00
Major leg amputations	4/94 (4.2)	0/183 (0.0)	0.01
Event rate (per 100 person-years)	3.9%	0.0%	–
Overall leg amputations	9/94 (9.6)	7/183 (3.8)	0.06
Event rate (per 100 person-years)	8.7%	2.5%	–

**Table 5:** Univariate and multivariate analysis for major leg amputations

	Univariate analysis HR (95% CI)	Multivariate analysis HR (95% CI)	*p* value
Smoking	1.27 (0.47–3.42)	2.01 (0.59–6.88)	0.26
Hypertension	0.77 (0.28-2.14)	0.57 (0.16-2.00)	0.38
Hyperlipidemia	0.62 (0.22–1.69)	0.16 (0.04–0.76)	0.02
Chronic kidney disease	1.09 (0.37–3.17)	0.78 (0.24–2.50)	0.68
Diabetes mellitus	4.88 (1.69–14.1)	9.95 (2.28–43.5)	0.002
Critical limb ischemia	8.54 (1.93–37.8)	10.5 (1.80–61.3)	0.009
Femoropopliteal anatomy	24.9 (1.46–425.7)	22.4 (1.00–518.7)	0.05
Stented lesion length	8.50 (1.93–37.4)	15.1 (2.0–114.1)	0.008
Chronic total occlusions	1.49 (0.54–4.10)	0.65 (0.15–2.79)	0.57
Stent grafts	7.29 (1.95–27.2)	7.33 (1.41–38.0)	0.02
Dual antiplatelet agents	1.05 (0.37–2.93)	1.57 (0.51–4.83)	0.44

## Discussion

Outcomes of this real-world, retrospective analysis suggest that stent thrombosis resulting in acute leg ischemia and potential limb loss occurs more often in the femoropopliteal segment and is significantly more frequent following covered nitinol stent (stent-graft) compared to bare nitinol stent. The herein cumulative stent thrombosis rate was 6.1%, which is in line with previously published data indicating that peripheral stent thrombosis is not a very rare phenomenon. In a similar setting, Vartanian et al. recently reported a nearly identical overall rate of 6.7% femoropopliteal stent thrombosis presenting with acute limb ischemia [10]. The authors also stratified their results according to type of stent, i.e., bare versus covered ones. According to the published subgroup analysis, there was no BNS thrombosis compared to nine thrombotic events resulting in ALI following CNS implantation (CNS 12 vs. 0% for bare stents; *p* < 0.05) [10]. These results are very similar to the herein presented findings of cumulative 13.8% covered stent thrombosis versus 2.2% in case of bare nitinol ones.

However, it must be stressed that the present results should be interpreted with caution as significantly longer, more complex lesions and significantly more CLI patients were included in the CNS arm. Still, multivariable analysis adjusted for confounders and actually identified stented lesion length as an independent predictor of major leg amputations, while CLI was correlated with both increased risk of stent thrombosis and major leg amputations. Notably, placement of covered stents was associated with an increased risk of both acute stent thrombosis (adjusted hazard ratio of 3.4 times) and future major amputations (adjusted hazard ratio of 7.3 times). The majority of our cases were prescribed on dual antiplatelet therapy (DAPT). However, we could not associate DAPT with a decreased risk of neither stent thrombosis nor major amputations, as previously reported in the literature [16]. Arguably, the Cox model may have limited power to detect any weak covariate relationships because of the relatively small number of event counts (17 stent thromboses and 16 major amputations, in total). In addition, we had no information available about the actual duration and patient compliance on DAPT. Dual antiplatelets and in particular patient responsiveness to Clopidogrel have been shown to significantly affect stent patency and long-term clinical outcomes and limb salvage [16–18].

Other studies investigating the use of covered stents in the femoropopliteal arteries have reported even higher rates of stent thrombosis. In particular, Golchehr et al. reported a 23% stent thrombosis rate within a 10-year period in a retrospective analysis of patients treated with ePTFE stent grafts in the superficial femoral artery (SFA) [19]. Of interest, 4-year results from the randomized comparison of percutaneous ePTFE stent-graft deployment versus prosthetic femoral-popliteal bypass demonstrated a high 36% rate of covered nitinol stent thrombosis with a mean time to thrombosis of 11.3 ± 6 months (range 1 h to 24 months) [20]. In the present study, CNS thrombosis occurred mainly within the first 6 months with a median time to thrombosis of around 1 month and a half. Stent oversizing >20% has been incriminated for covered stent failure and is a key technical consideration with VIABAHN stent use [21–23]. In the present study, the distribution of CNS diameters suggested more conservative sizing in case of covered stent placement in our cohort.

Another important finding of the present study is the incidence of bare nitinol stent thrombosis, which was found to be 1.4 events per 100 person-years and affected again exclusively the femoropopliteal artery. Of interest, the recently published ZEPHYR registry of the ZILVER-PTX paclitaxel-eluting nitinol stents in the femoropopliteal segment reported a 2% definite stent thrombosis rate at 1 year [24]. In the present series, we had one case of acute ZILVER-PTX thrombosis as well. Our findings are also congruent with the recent report by the XL-Pad registry on the incidence of femoropopliteal stent thrombosis. According to the XL-PAD findings, the rate of stent thrombosis was similar between drug-eluting and bare-metal stents (4.4 vs. 3.4%; *p* = 0.55), but significantly higher in the case of self-expanding covered stents as compared with bare-metal stents (10.6 vs. 3.4%; *p* = 0.02) [28].

To our knowledge, this is the first series reporting four individual cases of peripheral bare nitinol stent thrombosis resulting in limb-threatening acute ischemia. Notably, all four cases were successfully treated with either percutaneous catheter-directed thrombolysis or bypass surgery avoiding major amputation. On the contrary, nearly a third of the herein reported CNS thromboses (4 out of 13) resulted in major amputation compared to none from the bare nitinol group. This could be attributed to the fact that CNS thrombosis would be similar to surgical bypass thrombosis from a pathophysiological point of view. In the absence of adequate collateral arterial network to support foot perfusion, especially in cases of long infrainguinal covered stents, thrombosis may manifest as acute limb-threatening ischemia and urgent thrombolysis or bypass surgery may be the only effective means of treatment. Hence, ischemia following CNS thrombosis may develop clinically in a more abrupt and pronounced way putting the foot at risk of amputation, and its management may be technically more demanding, because of the PTFE excluding collateral networks especially in cases with poor foot run-off. Treatment of peripheral stent-graft thrombosis with transcatheter thrombolysis and/or mechanical thrombectomy has been reported in the literature with success rates ranging from 49 to 95%, while in cases of failed lysis/thrombectomy, surgical bypass has been a valid alternative in order to prevent imminent amputation but unfortunately not always successfully [19, 20].

We have also identified critical limb ischemia as an independent predictor of stent thrombosis and not surprisingly limb loss as well. A possible explanation could be that the complexity of arterial disease in terms of lesion length, severe calcifications and increased platelet reactivity noted in CLI patients may have contributed to the increased risk of stent thrombosis [17]. Moreover, progressive atherosclerotic disease affecting the distal and/or proximal stent landing zone could be another factor contributing to stent thrombosis especially in cases of covered stents, while thrombogenic intramural hematomas following stenting of long arterial subintimal dissection could be another possible mechanism for early bare-metal stent thrombosis. Diabetes (HR 9.9; *p* = 0.002), critical leg ischemia (HR 10.5; *p* = 0.009) and long lesions (HR 15.1; *p* = 0.008) were also found to be highly significant predictors of major limb loss as previously reported in the literature [24–27].

Limitations of this study include its single-centre, retrospective design that would account for the heterogeneity noted in the baseline demographic and procedural variables between the two stent cohorts. There is also an inherent operator selection bias because of more covered stent use in long femoropopliteal occlusions treated with a subintimal technique. Moreover, although hospital files were thoroughly searched, some cases of stent thrombosis and/or ALI might have been overlooked or may have developed in a more clinically subtle fashion and have been considered as chronic stent re-occlusions. However, similar to the ARC classification system of coronary stent thrombosis [13], we defined definite peripheral stent thrombosis as imaging confirmed stent thrombosis presenting with acute limb ischemia symptoms. The present study may also be underpowered in the aortoiliac vessels, considering the very low frequency of the phenomenon in that particular anatomy. Nonetheless, until today this is the largest series reporting the incidence and key risk factors of peripheral stent thrombosis adding valuable data about the potential clinical implications of early or late stent failure in the femoropopliteal segment.

In conclusion, peripheral artery stent thrombosis is an infrequent but truly serious phenomenon that may present with acute limb ischemia at risk of limb loss. Placement of long femoropopliteal covered nitinol stents is associated with an increased incidence of acute stent thrombosis and ensuing major amputation in particular. Risks are significantly lower in the aortoiliac vessels and with the use of bare nitinol stents. More studies are warranted considering the low frequency of the phenomenon.

## References

[1] Fowkes FG, Rudan D, Rudan I (2013). Comparison of global estimates of prevalence and risk factors for peripheral artery disease in 2000 and 2010: a systematic review and analysis. Lancet.

[2] Hirsch AT, Haskal ZJ, Hertzer NR (2006). ACC/AHA Guidelines for the Management of Patients with Peripheral Arterial Disease (lower extremity, renal, mesenteric, and abdominal aortic): a collaborative report from the American Associations for Vascular Surgery/Society for Vascular Surgery, Society for Cardiovascular Angiography and Interventions, Society for Vascular Medicine and Biology, Society of Interventional Radiology, and the ACC/AHA Task Force on Practice Guidelines (writing committee to develop guidelines for the management of patients with peripheral arterial disease)–summary of recommendations. J Vasc Interv Radiol.

[3] Moysidis T, Opdenplatz D, Kulendik V (2015). Percutaneous endovascular treatment of peripheral arterial disease in Germany. Cardiovasc Revasc Med.

[4] White CJ, Gray WA (2007). Endovascular therapies for peripheral arterial disease: an evidence-based review. Circulation.

[5] Pastromas G, Katsanos K, Krokidis M, Karnabatidis D, Spiliopoulos S (2014). Emerging stent and balloon technologies in the femoropopliteal arteries. Sci World J.

[6] Diamantopoulos A, Katsanos K (2014). Treating femoropopliteal disease: established and emerging technologies. Semin Interv Radiol.

[7] Katsanos K, Spiliopoulos S, Karunanithy N, Krokidis M, Sabharwal T, Taylor P (2014). Bayesian network meta-analysis of nitinol stents, covered stents, drug-eluting stents, and drug-coated balloons in the femoropopliteal artery. J Vasc Surg.

[8] Katsanos K, Tepe G, Tsetis D, Fanelli F (2014). Standards of practice for superficial femoral and popliteal artery angioplasty and stenting. Cardiovasc Interv Radiol.

[9] Spiliopoulos S, Theodosiadou V, Fragkos G (2014). Feasibility of endovascular recanalization of occluded infrapopliteal drug-eluting stents. J Endovasc Ther.

[10] Vartanian SM, Johnston PC, Walker JP (2013). Clinical consequence of bare metal stent and stent graft failure in femoropopliteal occlusive disease. J Vasc Surg.

[11] Holmes DR, Kereiakes DJ, Garg S (2010). Stent thrombosis. J Am Coll Cardiol.

[12] Siablis D, Karnabatidis D, Katsanos K (2009). Infrapopliteal application of sirolimus-eluting versus bare metal stents for critical limb ischemia: analysis of long-term angiographic and clinical outcome. J Vasc Interv Radiol.

[13] Luscher TF, Steffel J, Eberli FR (2007). Drug-eluting stent and coronary thrombosis: biological mechanisms and clinical implications. Circulation.

[14] Norgren L, Hiatt WR, Dormandy JA (2007). Inter-society consensus for the management of peripheral arterial disease (TASC II). J Vasc Surg.

[15] Sacks D, Marinelli DL, Martin LG, Spies JB, Society of Interventional Radiology Technology Assessment C (2003). Reporting standards for clinical evaluation of new peripheral arterial revascularization devices. J Vasc Interv Radiol.

[16] Katsanos K, Spiliopoulos S, Saha P (2015). Comparative efficacy and safety of different antiplatelet agents for prevention of major cardiovascular events and leg amputations in patients with peripheral arterial disease: a systematic review and network meta-analysis. PLoS ONE.

[17] Spiliopoulos S, Pastromas G, Katsanos K, Kitrou P, Karnabatidis D, Siablis D (2013). Platelet responsiveness to clopidogrel treatment after peripheral endovascular procedures: the PRECLOP study: clinical impact and optimal cutoff value of on-treatment high platelet reactivity. J Am Coll Cardiol.

[18] Spiliopoulos S, Kassimis G, Hatzidakis A, Krokidis M (2014). High on-treatment platelet reactivity in peripheral endovascular procedures. Cardiovasc Intervent Radiol.

[19] Golchehr B, Lensvelt MM, Fritschy WM (2013). Outcome of thrombolysis and thrombectomy for thrombosed endografts inserted in the superficial femoral artery for occlusive disease. J Endovasc Ther.

[20] McQuade K, Gable D, Pearl G, Theune B, Black S (2010). Four-year randomized prospective comparison of percutaneous ePTFE/nitinol self-expanding stent graft versus prosthetic femoral-popliteal bypass in the treatment of superficial femoral artery occlusive disease. J Vasc Surg.

[21] Lammer J, Zeller T, Hausegger KA (2015). Sustained benefit at 2 years for covered stents versus bare-metal stents in long SFA lesions: the VIASTAR trial. Cardiovasc Interv Radiol.

[22] Lammer J, Zeller T, Hausegger KA (2013). Heparin-bonded covered stents versus bare-metal stents for complex femoropopliteal artery lesions: the randomized VIASTAR trial (Viabahn endoprosthesis with PROPATEN bioactive surface [VIA] versus bare nitinol stent in the treatment of long lesions in superficial femoral artery occlusive disease). J Am Coll Cardiol.

[23] Saxon RR, Chervu A, Jones PA (2013). Heparin-bonded, expanded polytetrafluoroethylene-lined stent graft in the treatment of femoropopliteal artery disease: 1-year results of the VIPER (Viabahn Endoprosthesis with Heparin Bioactive Surface in the Treatment of Superficial Femoral Artery Obstructive Disease) trial. J Vasc Interv Radiol.

[24] Iida O, Takahara M, Soga Y (2015). 1-year results of the ZEPHYR Registry (Zilver PTX for the Femoral Artery and Proximal Popliteal Artery): predictors of Restenosis. JACC Cardiovasc Interv.

[25] Krankenberg H, Tubler T, Sixt S (2014). German multicenter real-world registry of stenting for superficial femoral artery disease: clinical results and predictive factors for revascularization. J Endovasc Ther.

[26] Sartori M, Conti E, Favaretto E, Frascaro M, Legnani C, Palareti G (2011). Thrombotic risk factors and cardiovascular events after endovascular intervention for peripheral arterial disease. Eur J Vasc Endovasc Surg.

[27] Conrad MF, Crawford RS, Hackney LA (2011). Endovascular management of patients with critical limb ischemia. J Vasc Surg.

[28] Banerjee S, Sarode K, Mohammad A (2015). Femoropopliteal artery stent thrombosis: report from the excellence in peripheral artery disease registry. Circ Cardiovasc Interv.

